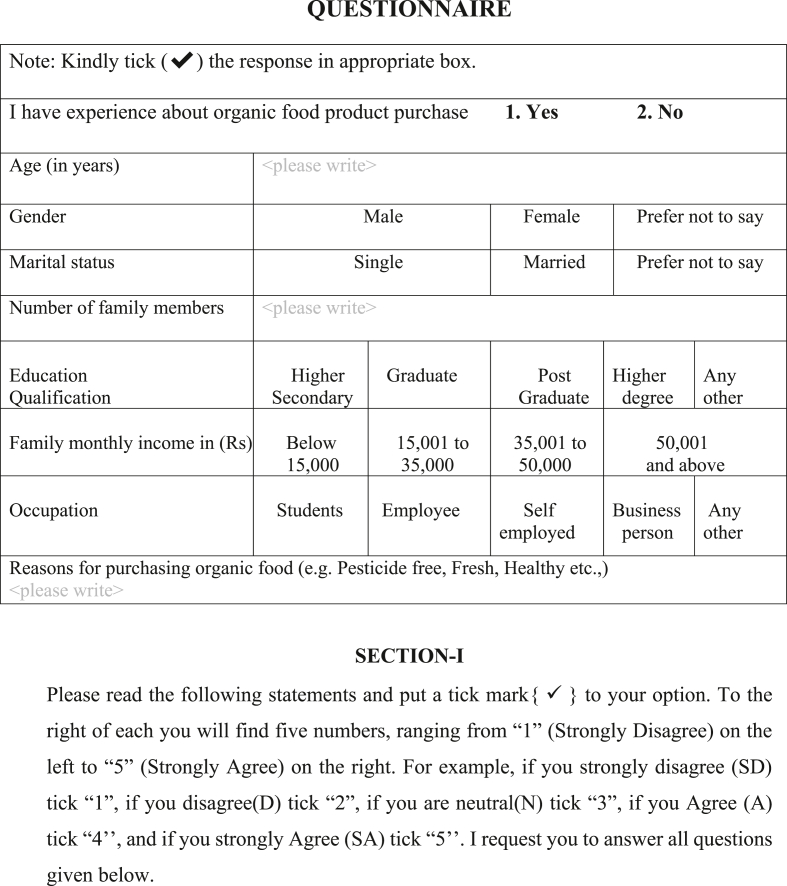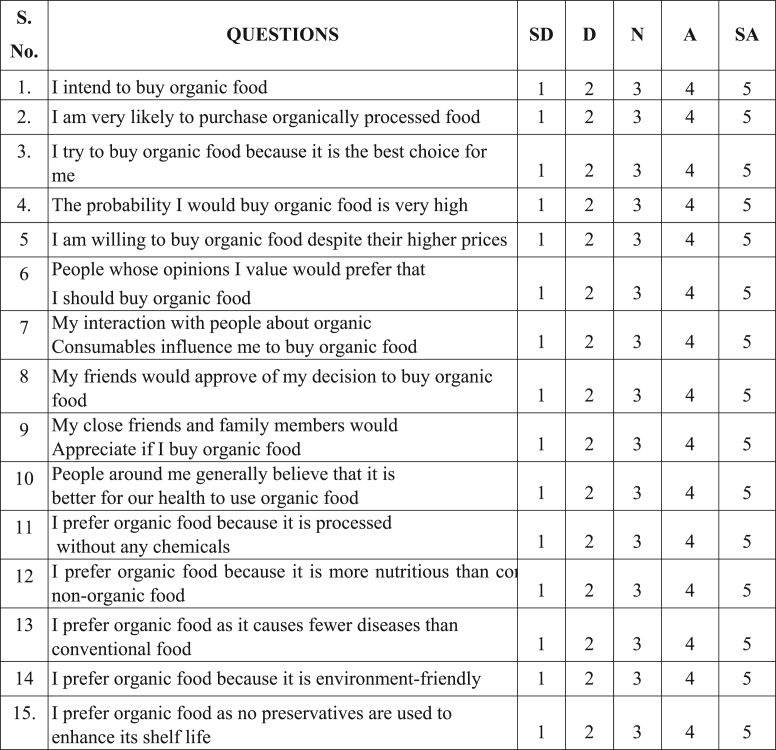# Does gender moderate the purchase intention of organic foods? Theory of reasoned action

**DOI:** 10.1016/j.heliyon.2022.e10478

**Published:** 2022-08-31

**Authors:** Raghava R. Gundala, Nishad Nawaz, Harindranath R M, Kirubaharan Boobalan, Vijaya Kumar Gajenderan

**Affiliations:** a University of Wisconsin - Parkside, 900 Wood Road, Kenosha, 53144, WI, USA; b Department of Business Management, College of Business Administration, Kingdom University, Bahrain; c School of Entrepreneurship and Management Studies, SRM University – AP, Amaravati, India; d SSN School of Management, SSN Institutions, Chennai, India; e Department of Commerce, Sir Theagaraya College, Chennai, India

**Keywords:** Organic food purchase intention, Subjective norm, Attitude, Gender, Multi-group moderation

## Abstract

**Purpose:**

This study examines the role of gender as a moderator on the relationships between subjective norm on attitude and purchase intention and attitude on purchase intention by using the Theory of Reasoned Action (TRA) among organic food consumers.

**Methodology:**

Data is collected using a crowdsourcing platform called Amazon's Mechanical Turk (MTurk). The respondents are organic food consumers (N = 633) from the US. The proposed model is tested using AMOS by covariance-based structural equation modelling and tested for multi-group moderation.

**Findings:**

The model is fit. The results of multi-group moderation show that gender moderates the two relationships: subjective norm on attitude and attitude on purchase intention, but not the third one, i.e., subjective norm and attitude. All the direct hypotheses are supported. This research found that males and females differ in purchasing intention toward organic food.

**Originality:**

This is the first study in the organic food context that tested subjective norm – attitude, attitude – purchase intention, and subjective norm—attitude using the theory of reasoned action.

## 1 Introduction

Gender is intertwined with all aspects of behavior and purchase behavior variations found to be substantial across gender regarding consumption (Kolyesnikova et al., 2009). Studies have reported that organic food consumption is growing (e.g., McFadden and Huffman, 2017), and young people and females purchase organic food more (Onyango Benjamin et al., 2007). Though organic food consumption increases over time, the literature found that gender is somewhat unclear regarding organic food consumption due to the inconsistent results reported in the studies. Researchers found females possess a greater consumption of organic food than males (e.g., Magnusson Maria et al., 2001; Rimal et al., 2005). On the contrary, Tsakiridou et al. (2008) reported that gender exhibit no difference in attitude and consumption of organic food. This study examines the moderating effect of gender on the following relationships; subjective norm—attitude, subjective norm—purchase intention, and attitude—purchase intent in the organic food domain. This is the study's contribution, and we followed the research gap identified by Nguyen The et al. (2017).

Existing literature identified several moderators in the relationship between attitude and purchase intention. The “attention to social comparison information” was found to moderate positively the relationships between attitude and purchase intention; and subjective norm and purchase intention (Chiou, 1998). The moderator “country of origin effect” positively moderates the relationship between attitude, subjective norm, and perceived behavioral control on purchase intention. Similarly, price sensitivity positively moderates the relationship between attitude - purchase intention and subjective norm - purchase intention (Hsu et al., 2017). Jain (2020) found that subjective norm negatively moderates the attitude-purchase intention link to luxury products. Self-transcendence values (consist of universalism and benevolence) moderate positively the relationship between attitude and purchase intention among organic food consumers (Zhou et al., 2013). Product knowledge negatively affects the relationship between subjective norms and purchase intention (Fu and Elliott, 2013). Finally, customer awareness positively moderates the relationship between attitude and purchase intent, subjective and purchase intention in an organic food setting. This study has samples from Pakistan, Turkey, and Iran (Asif et al., 2018).

In this vein, research has identified gender as a moderator in several consumer behavior domains. The relationship between gratitude and the amount spent is significant and positive for males and insignificant for females. Similarly, the influence of obligation on the amount paid is significant and positive for females and not significant for males (Kolyesnikova et al., 2009). These authors argue that males possess more product knowledge which will impact product gratitude and purchase. In contrast, in the case of females, the purchase is influenced through social aspects linked to obligation. Tikka et al. (2000) have shown the extent of knowledge an individual possesses about the environment will be based on their gender. Smith and Floro (2020) and Arganini et al. (2012) recognized gender could play an essential role in their food decisions and choices and reported that women compared to men were more conscious about body weight and were more likely to consume low-fat food. Lopez-Larson et al. (2011) used the fMRI (functional magnetic resonance imaging) technique and found men and women have different brain patterns when they were prompted to take high-calorie food (e.g. conventional food item) vs. healthy food (i.e. organic food). Hence, there are evidences to show that gender difference can effect green product consumption (Brough et al., 2016; Costa Pinto et al., 2014; Lee, 2009), organic food quality and purchase (Ureña et al., 2008), while other studies show that gender does not moderate the relationships between the following variables and behavioral intentions: perceived usefulness, perceived ease of use, subjective norm and perceived credibility (Kim, 2016). Further, research conducted in Europe has established that gender has a limited effect on the consumption of organic food (Gracia and de Magistris, 2008).

From the brief review of the relevant literature, the following emerges: One, the results obtained were mixed, which calls for greater research; our paper addresses this call (e.g., Ovseiko et al., 2016) (inclusion of gender in the assessment of impactful research). Second, to our knowledge, no study has employed the Theory of Reasoned Action (TRA) in conjunction with gender, and we fill this gap. Third, from the above review, gender moderates some relationships in the TRA, which may be a gap the literature is silent on. This gap is important to fill; if gender does not play a role, marketing strategies may be uniform for both. On the other hand, if gender does moderate some relationships in the TRA, this would suggest that practitioners need to adopt different marketing strategies for men and women, respectively.

In the USA, 20,000 (approx.) exclusive stores and 73% of conventional supermarkets sell organic products (Rimal et al., 2005). Further, the US is the largest market globally for organic food, with $45.2 billion in 2017 (Plaza, 2017). Hence, we test the hypothesized relationships with American consumers. We developed the TRA framework model to predict the outcome variable, i.e., purchase intention, subjective norm, and attitude as antecedents. Further, these relationships are tested using gender as a moderator in multi-group moderation. We found that the direct effect for all the relationships was positive and significant for males and females. Further, we found that the attitude – purchase intention and subjective norm – purchase intention relationships are moderated by gender.

## 2 Theoretical framework and hypotheses development

**Figure 1:**
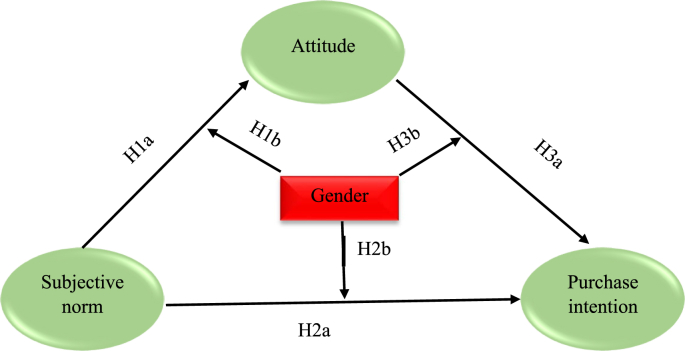
Hypothesized model.

This study used the theory of reasoned action (Fishbein, 1979). Individuals' decisions are due to volitional efforts for a particular behavior (Han and Kim, 2010). Individuals are rational and motivated while making decisions and choose a reasoned option among various available alternatives (Fishbein and Ajzen, 1977). The meta-study of Sheppard et al. (1988) confirmed the TRA's predictive power; it successfully predicts individuals' behavior in most cases, providing the behavior is volitional and not mandatory. In the context of TRA, purchase intention suggests the degree to which the consumers are ready or willing to purchase organic foods. The TRA has two components – the attitude (an individual's favorable or unfavorable assessment regarding a particular behavior) and subjective norm (perceived social pressure to perform or not to perform the behavior); both these affect behavioral intention, which is a proxy for actual behavior itself (Eagly and Chaiken, 1993). Likewise, our study uses subjective norm and attitude as predictors of purchase intention (Fishbein and Ajzen, 1977) (refer to Figure 1). Subjective norm positively influences behavioral intention (Teng and Wang, 2015).

We built our framework by using TRA. Further, for the moderation of gender, we utilized the general socialization theory and gender schema theory (Bem, 1981a). Thus, we have adopted two theories to describe the course of gender socialization, as there is no comprehensive, explanatory, and single theory, as suggested by Stockard (1999). Further to strengthen gender difference, we propose the expectations states theory (Ridgeway and Bourg, 2004) argues that gender shows variation in social behavior and considers gender a scheme of social outcomes inequality.

### 2.1 Subjective norm, attitude on purchase intention

Subjective norm is defined as the effect of “perceived social pressure to perform or not perform the behavior” (Ajzen, 1991 p. 188). The subjective norm is an individual belief towards a particular behavior. This behavior will be based on how the reference group views if they engage in that mentioned behavior. Prior studies in organic consumerism have established the relationship between subjective norms and attitude (Chang, 1998; Tarkiainen and Sundqvist, 2005). Al-Swidi et al. (2014) found a significant association between subjective norm and attitude. Teng and Wang (2015) suggest that an influencing role of others (i.e., subjective norm) can influence individual attitudes. Hence, we hypothesize:

H1a The subjective norm positively influences attitude towards organic food.

Subjective norm is perceived social pressure or influence by which a person might indulge in a specific behavior (Ajzen and Driver, 1991). People are inclined to behave in a particular way if they perceive that important persons around them will approve of their behavior. Thus, social influence encompasses how individuals modify their behavior to meet demands. The social influence is similar to the subjective norm (White et al., 2009). The meta-study of (Lockheed, 1985) and other studies (e.g., Schneider and Cook, 1995; Wagner et al., 1986) showed that males have more social influence than females. This is supported by gender expectation theory (Ridgeway and Bourg, 2004), which argues that gender affects the significance of gender as a status attribute. The lower status of women comparing men was highlighted during interactions between them. Thus men found more social influence than women (Carli, 2001). Furthermore, males possess a higher level of the subjective norm when compared to females (Ajzen, 2015), thus leading to a higher level of attitude for men. Therefore, we hypothesize:

H1b The subjective norm - attitude is moderated by gender, such that for males, the subjective norm – attitude path relationship is stronger vis-a-vis females.

Studies have found subjective norms can significantly affect behavioral intention (Teng and Wang, 2015). Ajzen (1991) describes social pressure exerted on individuals to engage in behavior that predicts behavioral intention. Prior studies (e.g., Dean et al., 2008; Laureti and Benedetti, 2018; Nejad et al., 2004) on organic consumerism have found that the relationship between the subjective norm and purchase intention of organic food is significant and positive. Hence, we hypothesize:

H2a The subjective norm positively influences the purchase intention of organic food.

Subjective norm is defined as an individual perception that persons who feel important to them think they must perform that behavior (Fishbein and Ajzen, 1975). In green marketing, the influence of family members and friends was found to be determinants of the subjective norm. Therefore, in investigating the level of the subjective norm due to gender differences, it is worthwhile to examine the extent to which men/women can react to the information given by their referents. The women display greater “feminine” traits such as tenderness as per the Bem Sex-Role Inventory (BSRI) (Bem, 1981b) others' feelings comparing males (Rosenkrantz et al., 1968). In similar research, females used constructs associated with harmonious groups, relationships, and concern for general group unity (Skitka and Maslach, 1996). In general, women tend to rate the significance of pleasing others more likely than men (Miller, 2012). The literature review of Minton and Schneider (1985) reported that women are people-oriented while men are more self-confident and independent.

Research has indicated gender differences in food (e.g., Rappoport et al., 1993). Napolitano et al. (2010) reported that women had better preferences towards consuming organic food than men. The study of Rimal et al. (2005) argued that women frequently purchase organic food than men. The males look for intrinsic pleasure in food, while females are inclined to healthy food (Rappoport et al., 1993). Fagerli and Wandel (1999) reported that females were disposed more to changes in diet comparing men and had more knowledge of foods. The cognitive abilities, expectations, and beliefs are developed and altered due to social influences mainly by friends and family (Cheah and Phau, 2005). All the above arguments are supported by expectation state theory (Ridgeway and Bourg, 2004), which argues that gender possesses social behavior differences. Hence, we say that females are more prone to influence others than men, purchasing food. Thus, we hypothesize:

H2b The subjective norm - purchase intention is moderated by gender, such that for females, the subjective norm – purchase intention path relationship is stronger vis-a-vis males.

### 2.2 Attitude and purchase intention

Attitude is defined as an individual's favorable or unfavorable assessment of a particular behavior (Ajzen, 1991). When the consumer attitude is more favorable towards a product, their purchase intention will be stronger (Ajzen, 1991). Many studies performed in the organic consumers context also have confirmed the significant influence of attitude towards the purchase intention of organic food (e.g., Enax et al., 2016; Boobalan et al., 2021; Wang &Tsai, 2014). Hence, we hypothesize that:

H3a Attitude positively influences the purchase intention of organic food.

Males and females behave differently, and this is due to the distinct socialization process they undergo (Blocker and Eckberg, 1997). This is well supported by gender socialization theory, which argues that girls and boys go through different socialization processes from the initial childhood stage that helps develop different values and social expectations (Chodorow, 1978). The gender difference found in grocery shopping was higher for online shoppers than in stores (Saphores and Xu 2021). Gender moderates the relationship between perceived channel risk-consumer intention to switch, intentions arise due to price-search - consumer intention to switch, mobility - consumer intention to switch, delivery time differences - consumer intention to switch, among grocery shoppers (Handayani et al., 2020). For example, Denton et al. (2004) found that engaging in health-related behavior is higher for females than men.

Further, Zelezny et al. (2000), in their research study performed in fourteen countries, found that females have a robust environmental attitude than males. However, we propose that the relationship between attitude and purchase intention is greater for females than males (Teng and Wang, 2015). Based on these premises, we hypothesize:

H3b The attitude – purchase intention is moderated by gender, such that for females, the attitude – purchase intention relationship is stronger vis-a-vis males.

## 3 Methodology

### 3.1 Data collection

Amazon's Mechanical Turk (M Turk), a crowdsourcing platform, collected responses. Many studies (e.g., Ipeirotis, 2010) have suggested that the data from this platform is reliable. We paid US$ 0.2 for each respondent in exchange for the completion of the questionnaire. Further, our study is similar to other studies in consumer behavior research that have collected data from the M Turk platform (Cadario and Chandon, 2019; Coary, 2018). All the respondents were explained about the study and informed consent was obtained from all the respondents before administering the questionnaire. Our respondents are from the USA, and the sample size was 633. The questionnaire begins with a screening question, “Do you consume organic food regularly?” to select the respondents. Only if the respondents marked “yes,” do they get the chance to answer the other questions in the questionnaire. The scales used in this study are adopted from the existing literature (refer to Table 2) and measured using a 5-point Likert scale (1 = strongly disagree & 5 = strongly agree). Age and education were used as control variables suggested by other studies (e.g., Hansen et al., 2018). The respondents' demographic details are presented in Table 1.

**Table 1:** Demographic characteristics.

	Categories	Frequency
Gender	Male	243
	Female	387
	Prefer not to say	3
Marital status	Single	325
	Married	299
	Prefer not to say	9
Educational qualification	Higher secondary	116
	Graduate	272
	Post-graduate	98
	Higher degree	100
	Any other	47
Occupation	Employed	374
	Self-employed	114
	Business	17
	Student	38
	Any other	90
Average age (in years)	36.7	
Monthly Income (in USD)	450

**Table 2:** Descriptive statistics.

Variables name	Mean	Standard Deviation	Correlation		
			1	2	3
1. Attitude	3.87	0.93	1		
2. Subjective norm	3.61	1.06	0.591∗∗	1	
3. Purchase intention	3.55	0.85	0.678∗∗	0.591∗∗	1

∗∗ correlation significant at 0.01 level.

### 3.2 Data analysis

The descriptive data analysis is performed using SPSS software and presented in Table 2.

The study design is cross-sectional, so common method bias (CMB) assessment is necessary. To examine the influence of CMB, we performed two tests – the marker variable test (Harindranath et al., 2019; Lindell and Whitney, 2001) and the common latent factor model (Harindranath and Sivakumaran, 2021; Podsakoff et al., 2012) using AMOS. We introduced “age” as a marker variable in the structural model like the previous studies (e.g., Rakesh et al., 2017), as age is unrelated to other variables of our study. We found the results are similar with and without the marker variable. Similarly, the results with and without the common single factor are almost identical, indicating the lack of common method variance. Hence, common method bias is not much of an issue for our study.

#### 3.2.1 Assessment of measurement model

The model was estimated sequentially for the overall sample (i.e. both male and female) – first the measurement model and then the structural model as suggested by Anderson and Gerbing (1988) using maximum likelihood estimation available in AMOS software (Aziz and Chok, 2013). The measurement model was analysed for confirmatory factor analysis (CFA) using the maximum likelihood estimation method. The value of χ2 minimum per degrees of freedom (CMIN/DF) is l.44, less than the cut-off value (<3); goodness-of-fit index (GFI) = .99 (cut-off value is > .95T to 1); comparative fit index (CFI) = .99 (cut-off value is > .95 to 1); normed fit index (NFI) = .99 (cut-off value >.95 to 1); incremental fit index (IFI) = .99 (cut-off value >.95 to 1); Tucker Lewis fit index (TLI) = .99 (cut-off value is >.95 to 1); standard root mean square residual (SRMR) = .017 (cut-off value is <.04); root mean square error of approximation (RMSEA) = .027 (cut-off value is <.06). The values of CMIN/DF, GFI, CFI, TLI and RMSEA are within the threshold limit suggesting that the CFA model fit is excellent (Hu and Bentler, 1999).

As shown in Table 3, the constructs of composite reliability are in the range of 0.77–0.91 for the entire sample, indicating that the constructs possess reliability. The values of items loadings are in the range of 0.928 (max) to 0.644 (min) and are significant (We dropped 1 item in purchase intention, two items in the subjective norm, and two items in attitude due to inadequate item loadings). The average variance extracted (AVE) for the three constructs is in the range of (0.53–0.72). It is above the cut-off value of 0.5, which presents the existence of convergence validity. The discriminant validity is assessed using the Fornell and Larcker (1981) method. The square root of AVE values is less than the inter construct correlation (refer to Table 5), indicating discriminant validity. In a nutshell, the constructs possess reliability and validity.

**Table 3:** Measurement model results.

Constructs (Authors)	Items loading Overall/Male/Female	CR Overall/Male/Female	AVE Overall/Male/Female
***Purchase Intention (PI)*** (Han et al., 2010)			
I intend to buy organic food (**PI1**)	0.81/0.84/0.79	0.91/0.92/0.91	0.72/0.74/0.72
I am very likely to purchase organically processed food (**PI2**)	0.77/0.78/0.77		
The probability I would buy organic food is very high (**PI3**)	0.58/0.60/0.57		
I try to buy organic food because it is the best choice for me (**PI4**)	0.78/0.72/0.82		
***Subjective norm (SB) (Armitage et al., 1999)***			
People whose opinion I value would prefer that I should buy organic food (**SB1**)	0.80/0.83/0.78	0.77/0.79/0.76	0.53/0.57/0.52
My interaction with people about organic consumables influences me to buy organic food (**SB2**)	0.78/0.80/0.76		
My friends would approve of my decision to buy organic food (**SB3**)	0.89/0.90/0.89		
***Attitude (ATT) (Wang et al., 2013)***			
I prefer organic food because it is processed without any chemicals (**ATT1**)	0.73/0.78/0.70	0.83/0.80/0.84	0.62/0.57/0.63
I prefer organic food because it is more nutritious than non-organic food (**ATT2**)	0.91/0.90/0.91		
I prefer organic food as it causes fewer diseases than conventional food (**ATT3**)	0.86/0.84/0.87		

CR is composite reliability and AVE is average variance extracted.

#### 3.2.2 Assessment of structural model

The structural model was estimated for complete samples and the results are: CMIN/DF = 1.444 (cut-off value is < 3); GFI = 0.987 (cut-off value is > 0.95); CFI = .996 (cut-off value is > 0.95 to 1); NFI = 0.998 (cut-off value is > 0.95 to 1); IFI = 0.996 (cut-off value is > 0.95 to 1); TLI = 0.995 (cut-off value is > 0.95 to 1); SRMR = 0.0168 (cut-off value is <0.04) and RMSEA = 0.027 (cut-off value is <0.06). The values of RMSEA, CFI, TLI, GFI and CMIN/DF are within the threshold values indicate that the structural model is good fit (Hu and Bentler, 1999).

All the direct hypotheses were supported (refer Table 5) and the results are follows; relationship between subjective norm to attitude (H1a) is positive and significant (β = 0.687, p < 0.001); the relation between subjective norm and purchase intention (H2a) is positive and significant (β = 0.338, p < 0.001); and relation of attitude and purchase intention (H3a) is positive and significant (β = 0.502, p < 0.001). The indirect effect between subjective norm and purchase intention via attitude is significant for total, male, and female group samples (refer to Table 5). Since the direct and indirect effects are significant, suggesting the presence of partial mediation.

### 3.3 Moderation analysis

Further, we performed multi-group analysis using gender as a moderator by employing the maximum likelihood estimation available in AMOS software, and are presented in Table 5. The gender is coded as male = 1 and female = 2. We then performed the CFA for both the samples (males and females) and found the following results. The results are then given in the following order (first for males and then females in Table 3), CMIN/DF = 1.253/1.136; GFI = 0.972/.983; CFI = 0.995/.998; NFI = 0.976/0.985; IFI = 0.995/.998; TLI = 0.992/0.997; SRMR = 0.0221/0.0224; and RMSEA = 0.032/0.019. The CFA results indicate that the measurement model fits both the group samples. The AVE value for all the constructs is above 0.5 for both the sub-samples, confirming convergent validity in the group samples. To assess the discriminant validity, we used the Fornell and Larcker (1981) method for both the samples and found discriminant validity in both the group samples (refer to Table 4). The composite reliability for male and female sub-samples for the three variables are; subjective norm = 0.79/0.76; attitude = 0.80/0.84; and purchase intention = 0.92/0.91. Thus the constructs possess reliability and validity for both the group samples.

**Table 4:** Discriminant validity (for total, male and female sample).

Constructs	1	2	3
1. Subjective norm	**0.73/0.75/0.72**		
2. Attitude	0.68/0.70/0.66	**0.79/0.78/0.79**	
4. Purchase intention	0.68/0.80/0.61	0.73/0.72/0.74	**0.85/0.86/0.85**

The column heading is the square root of AVE given in bold and the remaining are correlation among constructs. The values are given in the order; total samples, male samples and female samples.

**Table 5:** Structural model results.

Structural paths	Overall sample	Estimate (β_1_) (Male)	Estimate (β_2_) (Female)	Difference in Estimates (β_1_- β_2_)	P value for difference
Direct effect					
SB→ATT	0.687∗∗∗ (H1a)	0.709∗∗∗	0.667∗∗∗	0.042	0.492
SB → PI	0.338∗∗∗ (H2a)	0.582∗∗∗	0.203∗∗	0.379	0.002
ATT→ PI	0.502∗∗∗ (H3a)	0.311∗∗∗	0.614∗∗∗	-0.304	0.017
Indirect Effect for overall, male and female samples					
SB→ATT→PI	0.232 [0.157,0.304]	0.232 [0.119,0.361]	0.473 [0.348,0.627 ]	-0.241 [-0.430,-0.061]	0.031
Total Effect = Direct effect (DE) + Indirect effect (IDE)					
Total effect = DE + IDE	0.811 [0.811,0.982]	0.845 [ 0.736,0.959]	0.707 [ 0.585, 0.848]	0.138 [ -0.041,0.311]	0.189

† ∗∗p < .010, ∗∗∗p < .001, [] lower and upper limit of 95% percentile method. SB is subjective norm; ATT is attitude and PI is purchase intention.

**Figure 2:**
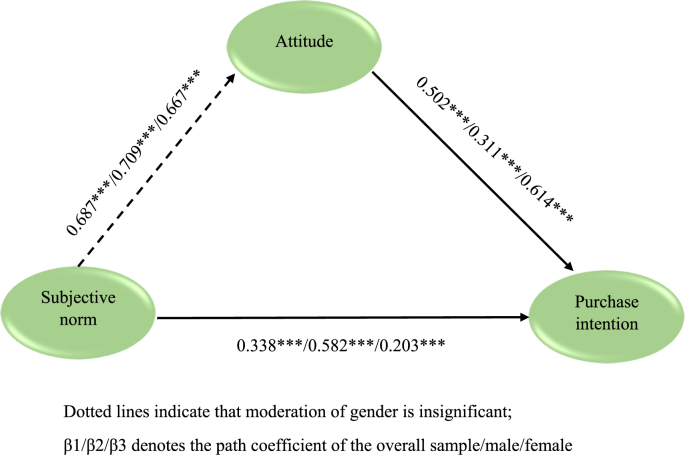
Structural model results.

As shown in Table 5, direct hypotheses were supported for male and female samples. The relationship between subjective norm to attitude is significant for both group samples; path coefficient is higher for males than females (β (males) = 0.709, p < 0.001; β (females) = 0.667, p < 0.001), supporting H1b, and difference between the path coefficient for male and female sample is insignificant (β difference = 0.042, p > 0.01). The hypotheses relating attitude and purchase intention is significant for male and female sub samples; the path coefficient is higher for females than males (β (males) = 0.311, p < 0.001; β (females) = 0.614, p < 0.001) supporting H3b, and significant across groups (β (difference) = 0.304, p < 0.001). The relationship between subjective norm and purchase intention is significant for the group samples; the path coefficient is higher for males than females (β (males) = 0.582, p < 0.001; β (females) = 0.203, p < 0.001) and group difference is also significant ((H2b) (β (difference) = 0.379, p < 0.001). Figure 2 represents the structural model results of this study, which comprises complete structural model analysis results of the overall sample, male, female group sample.

## 4 Discussion

The study developed a model with subjective norms, attitudes, and purchase intentions using the TRA framework. We found that subjective norm positively affects attitude. The result follows previous studies performed in the organic food context (Padilla Bravo et al., 2013; Teng and Wang, 2015; Yadav and Pathak, 2016). Further, the study found that attitude towards organic food influences purchase intention. The result is similar to earlier studies (e.g., Lee and Goudeau, 2014; Yeon Kim and Chung, 2011). Furthermore, we found that the subjective norm effect positively the consumer's attitude (towards organic food), which is in line with the findings of prior studies (Al-Swidi et al., 2014; Tarkiainen and Sundqvist, 2005). Further, we tested the same model with gender as a moderator. To our knowledge, no studies have used gender as a moderator in the organic food domain. Gender was used as a moderator between satisfaction and loyalty in a green restaurant study (Shapoval et al., 2018); Gender acts as a moderator between perceived inconvenience - purchase intention and environmental attitude – purchase intention using consumers of energy-efficient household electrical appliances (Nguyen The et al., 2017), and thus, our study adds to the existing literature. We found that the path coefficient is higher for male than female for subjective norm – attitude (β_males_ = 0.709; β_females_ = 0.667) and similarly for subjective norm – purchase intention relationships (β_males_ = 0.582; β_females_ = 0.203). Further, we showed that the strength of the relationship is higher for females than male samples, for attitude – purchase intention relationship (β_females_ = 0.614; β_males_ = 0.311), which is similar to the result obtained by Nguyen The et al. (2017) in energy-efficient appliances meant for the home. The relationship between subjective norm to attitude and subjective norm to purchase intention is invariant across gender, whereas the attitude to purchase intention relationship is not invariant.

## 5 Theoretical contribution

This study used gender as a moderating variable to test the relationships between subjective norm – attitude, attitude – purchase intention, and subjective norm - purchase intention, using the TRA framework in an organic food setting. We found that the relationships between the subjective norm - purchase intention and attitude - purchase intention vary across males and females. Chen et al.'s (2014) study is the only study to our knowledge that gender moderates the relationship significantly between attitude and purchase intention (negatively) in the organic food context. Our study differs from the above. We built a model and estimated using multi-group analysis using the SEM approach. Respondents were from the USA (individualistic culture). In contrast, the study (Chen et al., 2014) used a regression approach, and the respondents are from China (collectivistic culture). This is an important contribution of this study. Further, this study has revealed certain differences between males and females. This is similar to many gender difference studies; for example, gender differences occur in product preferences (Croson and Gneezy, 2009); use of technology (Chen, 1986), advertisement strategy (Brunel and Nelson, 2003). In this stream, we found that attitude matters more for males than females in predicting the organic food purchase intention (β_females_ = 0.614; β_males_ = 0.311); similarly, the subjective norm effect more for females (than males) for purchase decision of organic food (β_females_ = 0.614; β_males_ = 0.311); and path coefficient is higher for male than female for subjective norm – attitude relationship (β_males_ = 0.709; β_females_ = 0.667).

## 6 Marketing implications

The study findings can help US organic food firms (e.g., Costco) and others. Broadly, the study advocates that gender is associated with organic food consumption. Thus, it will assist in developing the marketing strategy. The firm needs to create a marketing strategy that focuses on gender. Specifically, the marketing strategy can contain some elements on gender. For example, the advertisement design can develop a story that portrays a male getting influenced due to societal pressure that further positively affects the purchase of organic food. Male consumers (more than females) look for society's approval (i.e., subjective norm) to purchase organic food as males' path coefficient is stronger. For example, social determining factors such as family, culture, and peers can be incorporated while designing the advertisement for male consumers, which can help bring sales.

Similarly, an advertisement design can show the attitude of a female (possibly a concern for the environment) and how that influences her purchase decision of organic foods. Hence, this study suggests that the firms actively consider consumers' gender-based segmentation. Further, this study found that attitude is higher for females (than males) in determining the organic food purchase, suggesting that firms concentrate more on the female population to increase their market share and profitability. This is demonstrated by Organic valley (a firm based in the USA), which portrays the attitude of women towards organic foods in their advertisements. It is highly successful as it was the first organic-only firm to cross USD 1 billion as turnover in 2015. The earlier studies of Lee (2009) and Schahn and Holzer (1990) also found that women show a high positive attitude towards environmentally friendly products in comparing men. Therefore, an essential ingredient of such a promotional campaign is to provide more knowledge about organic food, as knowledge helps form individual (female) attitudes (Argyriou and Melewar, 2011), which possibly influences the purchase of organic food.

## 7 Managerial implications

Apart from extending extant theory, this work also has tips for practitioners. First, we found that subjective norms directly affect purchase intention and attitudes (which drive purchase intention). Hence, in general, organic food companies may stress social norms (e.g., trendy and cool) rather than personal beliefs, which are the building blocks of attitudes (e.g., organic food is pesticide-free). Second, while organic food firms may also mention personal beliefs, they may stress social norms more generally, with some exceptions as delineated below.

Third, we found that attitudes play a more critical role for women than men. Therefore, while targeting women, organic food companies may stress personal beliefs (e.g., organic food is eco-friendly) to a greater extent rather than social norms—for example, the advertisement of Nature's Miracle and Nature's Path. Fourth, results showed that social norms mattered more for males vis-à-vis females. Hence, it is imperative that organic food brands showcase social norms, rather than personal beliefs, when targeting male consumers—for example, the Organic valley advertisement. Finally, organic food offers several benefits to consumers and the environment alike. However, the quantity of conventional food consumed continues to be much higher than that of organic food (i.e., only 5.5% of the retail food market in the USA) (Siegner, 2019). Extant literature mentions several reasons for this: affordable price, high yield, not implementable is a densely populated emerging country (Gomiero, 2018).

Apart from these, one additional explanation could be that organic food marketing is improper; typically, companies in this sector take a one-size-fits-all approach to organic market food. Our work suggests that a more nuanced approach is needed. Other industries like the car industry, the fitness industry, and the excellent consumer industry have different approaches for men and women. The organic food industry needs to cue these sectors and follow suit, i.e., have different targeting of men and women. For example, the Penny organic food advertisement portrays both males and females (Penny, 2021).

## 8 Limitations and further studies

Though the study has contributions, it has some limitations too. First, our study used the TRA as the conceptual framework. Future studies can use the theory of planned behaviour and extended theory of planned behaviour to understand purchase intention. Second, there are many possibilities for several studies across countries. The literature found the difference is substantial between similar cultures. For instance, gender does not influence studies performed in Europe, but education, age, and household size (Gracia and de Magistris, 2008), whereas gender, is critical in the US (Kolyesnikova et al., 2009). Third, the respondents of this study are from a developed country (i.e., the USA). Future researchers can test the model in an emerging market like India or compare developed vs. developing countries. For instance, Boobalan and Nachimuthu (2020) have performed a multi-group study comparing the samples of India and the USA and found significant variations in consumer perceptions regarding organic foods.

Further, future research can use gender trait difference as a moderator instead of gender (Kolyesnikova et al., 2009). The education level is a significant moderator of purchase intention of organic food (Gracia and de Magistris, 2008). Still, a comparison study between a developed country and an emerging market could be interesting.

## Declarations

### Author contribution statement

Raghava R. Gundala: Performed the experiment; Analyzed and interpreted the data; Wrote the paper.

Nishad Nawaz: Conceived and designed the experiments, Performed the experiment; Analyzed and interpreted the data; Wrote the paper.

Harindranath R M: Conceived and designed the experiments; Contributed reagents, materials, analysis tools or data; Wrote the paper.

Kirubaharan Boobalan: Conceived and designed the experiments; Contributed reagents, materials, analysis tools or data; Wrote the paper.

Vijaya Kumar Gajenderan: Analyzed and interpreted the data; Contributed reagents, materials, analysis tools or data; Wrote the paper.

### Funding statement

This research did not receive any specific grant from funding agencies in the public, commercial, or not-for-profit sectors.

### Data availability statement

No data was used for the research described in the article.

### Declaration of interest’s statement

The authors declare no conflict of interest.

### Additional information

No additional information is available for this paper.

## Figures and Tables

**Figure:**